# Apoptosis and Autophagy, Different Modes of Cell Death: How to Utilize Them to Fight Diseases?

**DOI:** 10.3390/ijms241411609

**Published:** 2023-07-18

**Authors:** Krisztina Takács-Vellai

**Affiliations:** Department of Biological Anthropology, Eötvös Lorand University, 1053 Budapest, Hungary; krisztina.takacs@ttk.elte.hu

A careful balance between cell death and survival is of key importance when it comes to the maintenance of cellular homeostasis. Although multiple pathways can lead to different forms of cell death, early studies focused on macroscopic morphological alterations and distinguished only three different types of cell death: apoptosis (type I), autophagy (type II), and necrosis (type III).

Over the past decades, a large body of experimental evidence has been accumulated, resulting in a detailed characterization of many genetically regulated processes which serve to eliminate irreversibly damaged or harmful cells. Today, more than a dozen different cell death modalities are known [1]. As novel factors regulating and orchestrating cell death processes are still being characterized, the Nomenclature Committee of Cell Death has defined cell death entities on a molecular and functional (rather than morphological) basis [2].

In this Special Issue, we focus on apoptosis and autophagy. Both processes belong to regulated cell death events, and act as highly synchronized mechanisms in cellular homeostasis.

**Figure 1:**
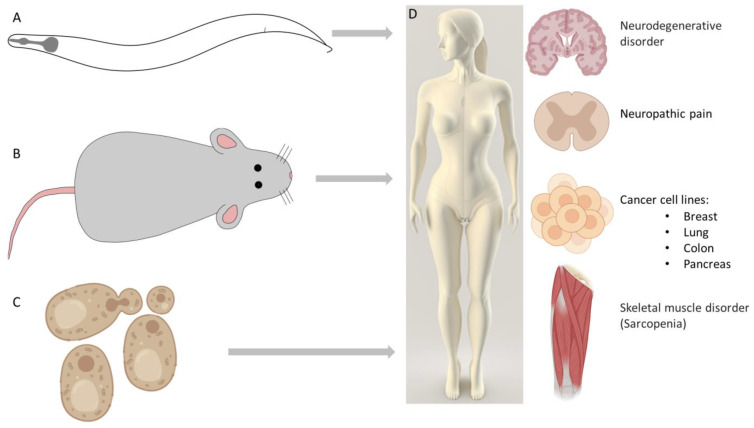
Model organisms played a significant role in uncovering cell death mechanisms. Abnormal regulation of apoptosis and autophagy is linked to numerous diseases. (**A**) The core apoptotic pathway was identified in the worm [22]. (**B**) Studies on mice contributed to the discovery of interleukin-1β-converting enzyme (ICE) caspases [23]. (**C**) Genetic regulation of autophagy was discovered in yeast [9]. (**D**) Some diseases related to abnormally regulated autophagy are discussed in this Special Issue: enhancing autophagic activity is an efficient tool to treat neurodegenerative disorders [14]. To prevent or treat sarcopenia, increasing mitophagy is a promising target [15]. Increased autophagic activity may also be useful to attenuate neuropathic pain behavior [17]. In cancer-related processes, autophagy plays dual roles. In this Special Issue, different treatments influencing autophagic activity and mitophagy were performed on breast, lung, pancreas, and colon cancer cell lines [19,20]. An illustration of this figure was partly created using BioRender.com (accessed on 2 March 2023).

Apoptosis is a caspase-dependent programmed cell death. Importantly, cells dying via apoptotic mechanisms do not induce inflammation, since cell debris are rapidly removed by phagocytes. Thus, three main apoptotic signaling pathways have been distinguished: the extrinsic (death receptors), the intrinsic (mitochondrial), and the perforin (granzyme) pathways [1]. While the extrinsic pathway is a result of a recent evolutionary development as it is only present in vertebrates, the intrinsic pathway appears to be ancient, and its components exist in all metazoans [3]. The review of Izadi et al. in the current issue gives an insight into the apoptosis research conducted on model organisms, especially the nematode *Caenorhabditis elegans*, for which a series of excellent genetic screens were performed to uncover the conserved core signaling pathway of intrinsic apoptosis [4] (Figure 1).

Cytochrome C (cytC) release from the mitochondria to the cytoplasm is a key event of intrinsic apoptosis in higher eukaryotes. Poulaki and Giannouli summarize our knowledge about changes in mitochondrial lipid composition leading to apoptotic facilitation. Cardiolipin (CL) plays a key role in cytoplasmic cytC release, as CL directly interacts with cytC and docks it to the mitochondrial inner membrane. CL translocation to the outer mitochondrial membrane is an established hallmark of upcoming cytC release and cell death, although the exact mechanisms that drive the flipping of CL to the outer membrane remain to be determined [5]. Lipidomics is an emerging field in cancer biology, also in relation to apoptosis; alterations in membrane composition and fluidity have been observed in apoptosis-resistant tumor cell lines [6]. A better understanding of lipidomic changes may help in developing novel interventions that render resistant cells more susceptible to therapies.

The initiation and execution of apoptosis occur in three main steps: first, the cells undergoing cell death are specified; next, in the execution phase, apoptosis is activated and regulated; finally, dying cells are eliminated via phagocytosis. Lukácsi et al. (in this issue) reviewed and compared the elements of phagocytosis in mammals and the nematode. Phagocytic cells ensure two vital functions: they prevent the accumulation of cell corpses to avoid pathological inflammation, and maintain host defense by removing pathogens. Although the initial recognition of infection requires different mechanisms, the engulfment and elimination of dying cells are highly conserved between worms and mammals. Emerging data in both species show that the two phagocytic functions are interconnected; the defective clearance of apoptotic cells activates innate immunity [7].

Autophagy was originally characterized as a cell survival mechanism in response to starvation, but it is also known as a cell death modulator once the cellular stress becomes irreversible. Autophagy, the process of “self-eating”, refers to the sequestration of proteins and organelles by double-membrane vesicles and the subsequent degradation by the lysosomal system of the cell [8]. The genetic pathways involved in the execution of autophagy were identified in the model organism yeast *Saccharomyces cerevisiae* [9] (Figure 1). Upstream mechanisms, which regulate autophagy, are still under investigation, and, in this issue, Pan et al. discuss the mechanisms by which the Glycogen Synthase Kinase-3 (GSK-3) signaling pathway regulates autophagy [10].

Autophagy can be induced both in physiological and pathological circumstances. Autophagy is often termed as a Janus-faced process, as it either contributes to cell survival or leads to cell death depending on the conditions [11].

The abnormal regulation of cell death processes is linked to numerous diseases, such as cancer, degenerative, cardiovascular, and autoimmune diseases [1]. While increased apoptosis can lead to neurodegenerative and autoimmune disorders, its downregulation can assist tumor progression [4].

The role of autophagy in human diseases is generally complicated [12].

In neurodegenerative disorders, the neuronal accumulation of protein aggregates can be observed, which is caused by the failure of autophagy; thus, increasing autophagic activity is an efficient tool for fighting neurodegenerative diseases (Figure 1). Sirtuins are evolutionarily conserved deacetylases regulating metabolic and aging processes, and have been shown to regulate autophagy as well [13]. The vital role of sirtuins and the mechanisms through which they modulate autophagy are described in Naseer et al., focusing on the knowledge obtained using the *C. elegans* model [14].

Sarcopenia, a currently untreatable condition, refers to the loss of skeletal muscle mass and strength during ageing, and it is associated with the accumulation of dysfunctional mitochondria. Enhancing mitophagy (Figure 1), which is responsible for the elimination of damaged mitochondria, seems to be a promising target for preventing or treating sarcopenia in the elderly (reviewed in [15,16]).

Neuropathic pain is caused by damage to the somatosensory system. Recent studies have revealed that apoptotic and autophagic activities change in the injured nerve, dorsal root ganglia, and dorsal horn (Figure 1). The upregulation of autophagy promotes nerve regeneration through myelin clearance, and may inhibit neuropathic pain. Pain behavior may be further attenuated due to increased autophagic activity through the inhibition of proinflammatory cytokine activities (summarized in [17]).

In cancer-related processes, autophagy has two roles: on the one hand, it may suppress tumor progression through the elimination of oncogenic proteins and damaged organelles; on the other hand, autophagy often contributes to tumor progression and therapy resistance as an essential mechanism for tumor survival and growth under stress conditions [18]. Dankó et al. targeted metabolic rewiring, mammalian target of rapamycin (mTOR) hyperactivity, and mitochondrial functions through treating breast cancer cells using a rapamycin plus doxycycline combination. They observed that the combined treatment resulted in decreased tumor proliferation, did not cause apoptosis or necrosis/necroptosis, but led to the degradation of mitochondria and mitophagic activity [19]. In the current issue, Cha and colleagues reported the effect of 6-azauridine (6-AZA) on various human cell lines. The authors found that 6-AZA induces autophagy-mediated cell death in a p53- and AMP-activated protein kinase (AMPK)-dependent manner [20].

The induction of apoptotic tumor cell death is a successful strategy that has been applied in cancer treatment for a long time [21]. Although autophagy acts as a double-edged sword during different stages of tumor progression, novel results derived from different tumor cell lines published in this issue [19,20] show that autophagy could be targeted in future cancer therapies as well. However, first, further investigations are needed to address important questions regarding autophagy and cancer therapy resistance, and the issue of autophagy and cancer stem cells; in addition, novel methods should be developed to track autophagy in patients [18].
